# Effectiveness, Engagement, and Safety of a Digital Therapeutic (CT-155/BI 3972080) for Treating Negative Symptoms in People With Schizophrenia: Protocol for the Phase 3 CONVOKE Randomized Controlled Trial

**DOI:** 10.2196/81293

**Published:** 2025-10-07

**Authors:** Shaheen E Lakhan, Cornelia Dorner-Ciossek, Olya Besedina, Faith Dickerson, Claudia Hastedt, Ridwana Isla, René S Kahn, Jean-Pierre Lindenmayer, Ruchi Mehta, Cassandra Snipes, Austin Speier, Wenbo Tang, Bailey Willis, Jamie Winderbaum Fernandez, Christoph von der Goltz, Abhishek Pratap

**Affiliations:** 1 Click Therapeutics Inc New York, NY United States; 2 Boehringer Ingelheim International GmbH Ingelheim am Rhein Germany; 3 Sheppard Pratt Health System Towson, MD United States; 4 Boehringer Ingelheim Pharmaceuticals Inc Ridgefield, CT United States; 5 Icahn School of Medicine at Mount Sinai New York, NY United States; 6 Department of Psychiatry New York University New York, NY United States; 7 IPTB Clinical Research Tampa, FL United States

**Keywords:** digital control, digital therapeutics, negative symptoms, psychosocial intervention, randomized clinical trial, schizophrenia, smartphone, patient-centricity

## Abstract

**Background:**

Negative symptoms of schizophrenia, such as lack of motivation, pleasure, social interest, and expression, are key contributors to functional impairments in people with schizophrenia. While psychosocial interventions have demonstrated efficacy, no Food and Drug Administration–approved pharmacotherapies exist specifically to target these symptoms. Evidence-based digital therapeutics (DTx) may offer novel, scalable treatment options to augment existing treatments.

**Objective:**

This article describes the study design and methods of a phase 3, multicenter, double-blind, randomized controlled study (CONVOKE). It aims to evaluate the effectiveness and safety of CT-155/BI 3972080 (CT-155), a smartphone-based DTx, as an adjunct to standard-of-care antipsychotic medication in adults with experiential negative symptoms of schizophrenia.

**Methods:**

Eligible participants were 18 years or older with a primary diagnosis of schizophrenia receiving stable antipsychotic medication for ≥12 weeks, scored ≥2 on average in at least 2 Clinical Assessment Interview for Negative Symptoms Motivation and Pleasure subscale (CAINS-MAP) domains, and were smartphone owners. Participants were randomized 1:1 to CT-155 (intervention arm) or a digital control app (control arm). CT-155 integrates aspects of multiple evidence-based psychosocial therapeutic techniques, incorporating principles of in-person psychotherapy aimed at targeting negative symptoms. Development of CT-155 was informed by patients during early clinical learning studies using earlier versions of the app. The digital control included elements of the disease educational components of CT-155 and daily digital check-ins. Participants were blinded to their assigned intervention. A blind-to-hypothesis was used so participants appropriately engaged with both apps. Accordingly, participants were informed that they would receive one of 2 interventions under investigation. Investigators, designated site personnel, and central raters were blinded throughout the study. The study comprised a 2-week screening period, 16-week active period, and a 4-week follow-up period. Change in experiential negative symptoms from baseline to Week 16 (primary end point) was assessed using CAINS-MAP (centrally rated). Other study end points included Clinical Assessment Interview for Negative Symptoms Expressivity, Positive and Negative Syndrome Scale, Personal and Social Performance Scale, Dysfunctional Attitudes Scale, Clinical Global Impressions-Severity, and Patient Global Impression of Improvement Scale. Frequency and severity of adverse events were also assessed, as well as engagement and adherence to either app.

**Results:**

Study enrollment began in March 2023 and was completed in January 2025. Overall, 457 participants were enrolled across 66 clinical study sites in the United States.

**Conclusions:**

We summarize an innovative trial design for CONVOKE, a phase 3 randomized controlled study aimed at assessing the effectiveness, engagement, and safety of CT-155 as an adjunct to standard-of-care for people with negative symptoms of schizophrenia. CONVOKE is the largest-to-date and most robust clinical trial evaluating the effectiveness and safety of a DTx in schizophrenia. The study protocol included a centrally rated primary end point (CAINS-MAP), blind-to-hypothesis, with an appropriately designed digital control.

**Trial Registration:**

ClinicalTrials.gov NCT05838625; https://www.clinicaltrials.gov/study/NCT05838625

**International Registered Report Identifier (IRRID):**

DERR1-10.2196/81293

## Introduction

Schizophrenia is a complex psychiatric condition with a large disease burden and is one of the top causes of disability globally, presenting considerable challenges for patients, caregivers, and health care systems [1-4]. In the United States, people living with schizophrenia have a severely reduced life expectancy (losing ≈28.5 years of life) [5] and significantly higher mortality rate [6] than the general population. The economic burden of schizophrenia is also substantial, with estimated costs exceeding US $340 billion in 2019 [7]. Symptoms of schizophrenia are categorized into 3 primary domains: positive, negative, and cognitive [8,9]. Negative symptoms are a major contributor to poor quality of life, frequently occurring early in the prodromal phase and often persisting throughout all stages of the illness [10-14]. Despite several clinical trials, no pharmacological treatment has demonstrated efficacy for negative symptoms [15-23], and to date, no medication has been specifically approved by the Food and Drug Administration (FDA) to target negative symptoms [24,25].

Negative symptoms are conceptualized as being either expressive or experiential: expressive negative symptoms include affective flattening (reduced emotional expression) and alogia (reduced verbal expression), while experiential negative symptoms (ENS) consist of asociality (lack of social interest), avolition (lack of goal-directed behavior), and anhedonia (inability to experience joy or pleasure) [10,26]. People living with schizophrenia report a significant impact of negative symptoms on their daily functioning, through loss of interest in social activities, diminished pleasure, and reduced motivation [27]. These symptoms not only affect people with schizophrenia, but also place a substantial burden on their families and the wider health care system [28]. Furthermore, ENS have been associated with poorer outcomes than expressive negative symptoms [29,30]. Recent findings from a workshop conducted as part of the FDA’s patient-focused drug development initiative [27,31], showed that patients and their caregivers have also expressed a need for new treatments to help meaningfully improve their quality of life (eg, by helping them enjoy daily activities, improving motivation, and making it easier to maintain relationships) [27].

Current schizophrenia treatment guidelines recommend a comprehensive and person-centered approach that includes pharmacological treatments (eg, antipsychotics) and evidence-based nonpharmacological treatments (eg, adjunctive psychosocial intervention) [32-34]. While many antipsychotics approved by the FDA for the treatment of schizophrenia help stabilize and manage positive symptoms, their effects on negative symptoms remain limited and inconsistent, with many individuals experiencing little to no meaningful improvement [10,33,35-37].

Research has demonstrated that psychosocial interventions may target negative symptoms by focusing on cognitive restructuring of dysfunctional beliefs [38-50]. These interventions aim to help revise internal motivation and pleasure seeking in people with schizophrenia, fostering a desire to pursue meaningful goals by reducing defeatist beliefs [42,51,52]. However, psychosocial interventions can be time-intensive, costly, and require specialized clinicians, who are not widely available [53-55]. Accordingly, there remains a critical need for innovative treatments that are not only effective and accessible, but also designed to navigate symptom-related challenges (eg, stigma and social isolation) and systemic health care barriers [53,56-63].

With many people living with schizophrenia having access to smartphones [64,65], evidence-based clinically evaluated digital therapeutics (DTx) can help expand the nonpharmacologic treatment options. Benefits of DTx include access to care anytime and anywhere, cost effectiveness, support during long wait times between referral and beginning in-person therapy, additional support between in-person therapy sessions, continuity of care, and anonymity, allowing people to avoid stigma or feelings of judgment [63,66-69]. Additionally, including patient perspectives during the development of a DTx may support the creation of treatments that can better assimilate into daily routines within the patient’s own environment. However, DTx are often perceived to have limitations, including concerns about the lack of rigorous and inclusive clinical trials [70]. In the context of schizophrenia specifically, additional challenges include difficulties with patient engagement and adherence, and barriers related to access to technology and the requirement of digital literacy to use these interventions effectively [71].

Early research studies have shown the promise of DTx for the treatment of symptoms of schizophrenia [72-74]; however, these findings should be interpreted in the context of their limitations, such as small sample size and single-arm design, which are common in exploratory research [72-74]. More recent randomized controlled trials have produced mixed results. For example, a larger study evaluating PEAR-004 found no statistically significant difference in the reduction of positive symptoms compared with a digital control after 12 weeks [75], despite an early single-arm study showing clinical effectiveness [76]. Similarly, the Actissist study reported no significant differences between the DTx intervention and a remote symptom monitoring app used as an active control [77]. These findings illustrate the methodological complexity of evaluating DTx, particularly when control conditions may themselves carry therapeutic benefit. They also underscore the importance of adequately powered, controlled trials with well-characterized digital comparators to establish confirmatory evidence, in addition to early-phase clinical learning studies.

To establish DTx as a new treatment modality in mental health, it is especially important to consider study design elements such as appropriate blinding of both participants and investigators to treatment assignments and blinding of participants to the study hypothesis. Blinding minimizes bias and promotes comparable levels of engagement across both treatments [78,79]. Furthermore, the FDA recommends including a digital control when possible, to mimic the actual intervention without providing therapeutic benefit [80], while still maintaining blinding and face-validity [78].

Accordingly, there is a critical need for robust, well-designed randomized clinical studies to rigorously evaluate the effectiveness and safety of DTx to the standards of pharmaceuticals. This is not only to generate the evidence required for FDA regulatory approval but also to support establishing DTx as a new treatment modality delivering safe and effective treatment to patients. To this end, we are conducting a large phase 3 randomized, controlled, double-blind, 16-week study (CONVOKE; NCT05838625) to evaluate the effectiveness and safety of CT-155/BI 3972080 (CT-155) in reducing ENS compared with a digital control. Briefly, CT-155 was developed as a smartphone-based DTx that provides aspects of psychosocial intervention techniques adjunctive to standard-of-care antipsychotic medication to treat negative symptoms of schizophrenia [74,81]. CT-155 was granted Breakthrough Device designation in December 2023 [82]. Here, we discuss the rationale and study design for the CONVOKE study, which refers to study protocol version 5.0 (dated September 2024).

## Methods

### Study Design

#### Overview

**Figure 1:**
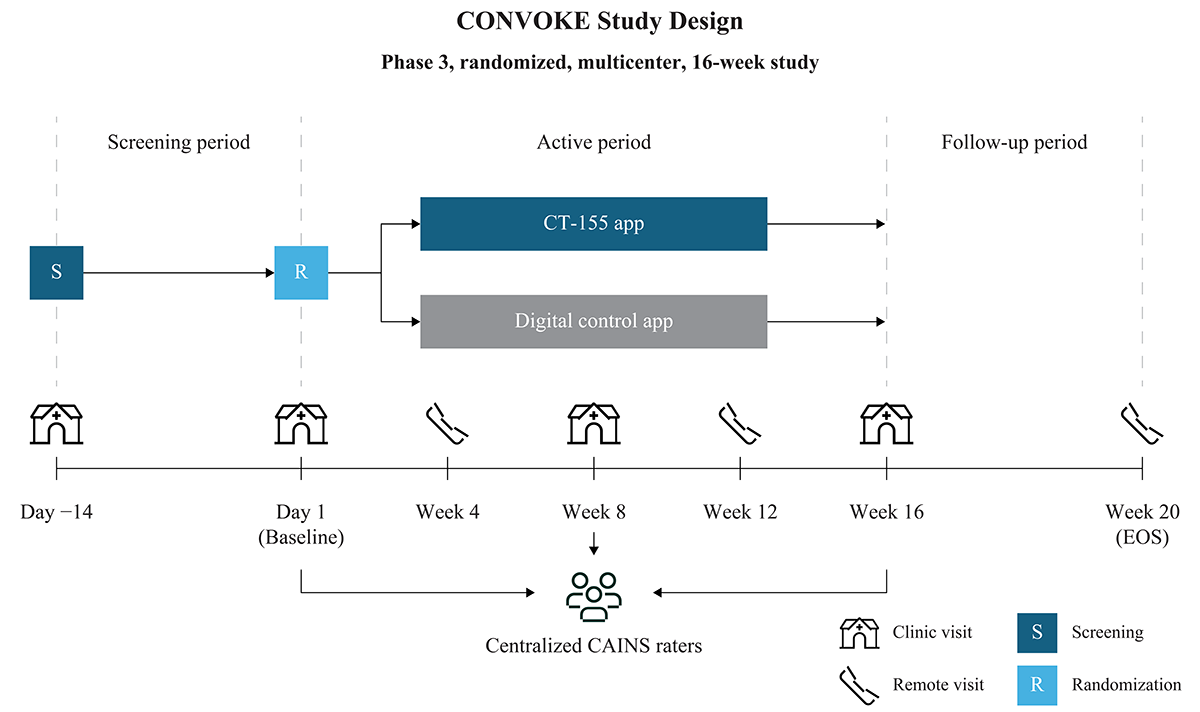
Study design. CAINS: Clinical Assessment Interview for Negative Symptoms; CT-155: CT-155/BI 3972080; EOS: end of study.

CONVOKE is a phase 3, multicenter, randomized, double-blind, parallel-group, controlled, 16-week study to evaluate the effectiveness and safety of CT-155 as an adjunct to standard-of-care antipsychotic medication in participants 18 years or older with ENS of schizophrenia (NCT05838625). Participants were enrolled from 66 of 82 activated study sites in the United States. The study, conducted between March 2023 and July 2025, consisted of a 2-week screening period, a 16-week active period, and a 4-week follow-up period (Figure 1). The study is reported in accordance with the SPIRIT checklist.

The study had 2 arms; CT-155 (active treatment) and digital control, which were masked as digital therapeutic A and digital therapeutic B, respectively, to maintain the blind-to-hypothesis (further details described below). Study interventions were activated following confirmation of eligibility during the baseline visit.

Eligible participants were randomized 1:1 on Day 1 using an interactive web response system to receive either CT-155 or the digital control app as the study intervention during the active period of the study.

#### Study Blinding

A blind-to-hypothesis design was used to help maintain face validity of the digital control app and adequate engagement with the study app in both arms [78]. Participants were blinded to the intervention assignment, as well as the study hypothesis. They were informed that they would receive one of the 2 digital interventions under investigation, both of which were called the “study app.” Study participants were told that both interventions may or may not be effective and that the study was comparing the effectiveness of the 2 interventions.

Investigators, designated site personnel, and central raters were also blinded to participants’ assigned study interventions throughout the course of the study. To maintain this blinding and mitigate the risk of blinded personnel being exposed to unblinded information, separate designated site personnel (ie, not investigator/rater) assisted with downloading, installing, and activating the study app and were responsible for conducting adherence checks. To further minimize potential bias, a blinded centralized rater team administered the primary outcome assessment, Clinical Assessment Interview for Negative Symptoms (CAINS).

#### Study App Procedures

**Figure 2:**
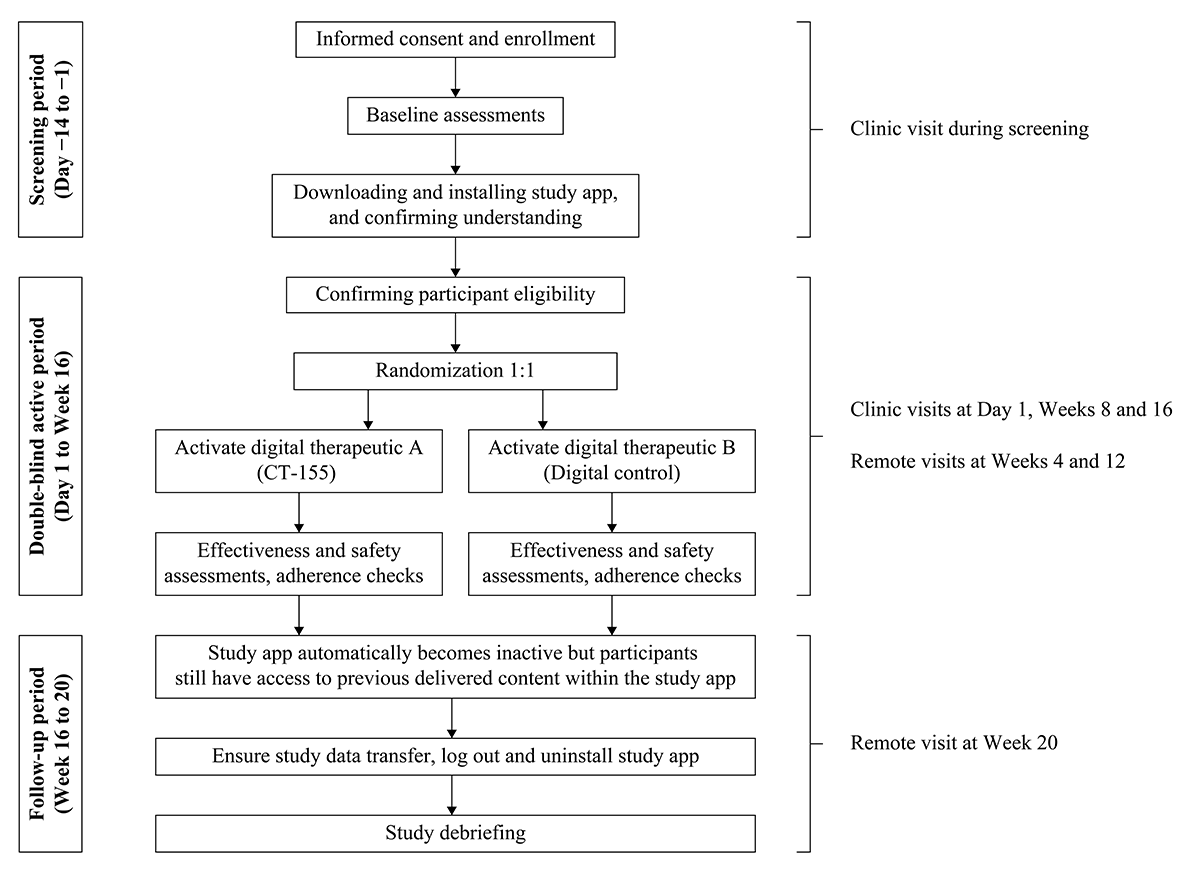
Study design flow and study app procedures in CONVOKE. CT-155: CT-155/BI 3972080.

During an in-person screening visit, designated site personnel assisted participants with downloading, installing, and activating their assigned study app, including account creation and account set-up. These site personnel were unaware of the individuals’ assigned intervention. The same app was downloaded for all participants, but was configured based on an access code to either CT-155 or the digital control. After the completion of Week 16, the study app automatically became “inactive” and stopped proactively engaging the participant with therapeutic or control content. However, participants retained access to previously delivered content within the study app during the 4-week follow-up period. During Week 20 or upon discontinuation, designated unblinded site personnel instructed participants to log out of and uninstall the study app. The study procedures are presented in Figure 2. The study app automatically transferred data collected throughout the study. To reduce the risk of data transfer issues the study app displayed an error message if the participant had not connected to the internet for 2 days. Furthermore, consistent reminders were shared with the site to remind the participants to remain connected to Wi-Fi. Participants had access to human technical support throughout the study period. If participants experienced issues with the study app, they could contact support via email or telephone. Support staff were trained to troubleshoot common issues, guide participants through resolution steps, and, if needed, escalate more complex cases to designated escalation personnel who investigated the issue and provided updated instructions or workarounds. These cases were reviewed and resolved collaboratively, and participants were followed up with until the issue was addressed.

### Study Participants

Eligible participants were 18 years or older, fluent in written and spoken English, and had a primary diagnosis of schizophrenia as defined in the *DSM-5* (*Diagnostic and Statistical Manual of Mental Disorders* [Fifth Edition]) for ≥6 months prior to the screening visit. Participants were required to have been on a stable dose of antipsychotic medications for ≥12 weeks prior to randomization, to have obtained an average score of ≥2 (moderate to severe) in at least 2 of the 3 Clinical Assessment Interview for Negative Symptoms Motivation and Pleasure subscale (CAINS-MAP) domains (Social, Work or Recreational) at both the screening visit and at baseline, and to be the sole user of a smartphone. Individuals were excluded if they had a *DSM-5* diagnosis for schizophreniform or schizoaffective disorder, or were receiving or had received psychotherapy. Full eligibility criteria are presented in Multimedia Appendix 1.

### Ethical Considerations

This study was conducted in accordance with the Declaration of Helsinki and the Good Clinical Practice Guidelines. The study protocol was approved by the Institutional Review Boards prior to study initiation. Institutional Review Board services were provided by Advarra (registration number Pro00065686). All participants were asked to provide written informed consent prior to starting the study and were informed that their participation was voluntary. All information collected during the study was considered confidential and was only used in accordance with the protocol, and participants were assigned a unique identifier by the sponsor at screening. Participants were offered up to US $45-US $200 and US $25-US $136 per in-person and remote visit, respectively, based on study center locality. While participants were compensated for their time attending site visits, they were not compensated for completing daily in-app activities. An independent Data Safety Monitoring Board reviewed safety events throughout the study period.

### Intervention

CT-155 is an investigational smartphone-based DTx being developed for use as an adjunct to standard-of-care antipsychotic medications in adults with negative symptoms of schizophrenia. CT-155 was designed based on the underlying principles of face-to-face treatment to provide maximal support to people living with schizophrenia [83-86]. By integrating multiple components of evidence-based psychosocial therapeutic techniques in a patient-centered manner, CT-155 aims to address the dual challenge of accessibility and psychosocial therapy use for people with negative symptoms living with schizophrenia.

**Figure 3:**
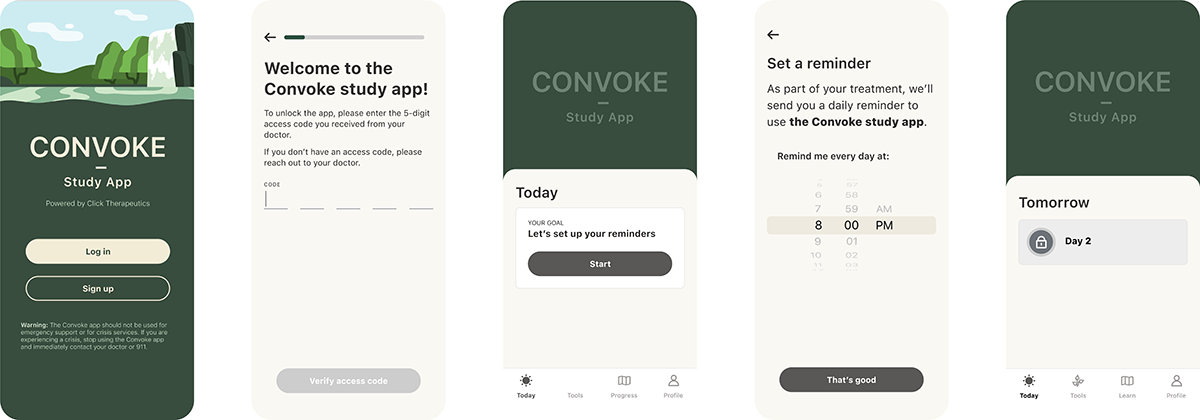
Example screenshots of the CT-155 study app and digital control app representing the login and onboarding experience. CT-155: CT-155/BI 3972080.

**Figure 4:**
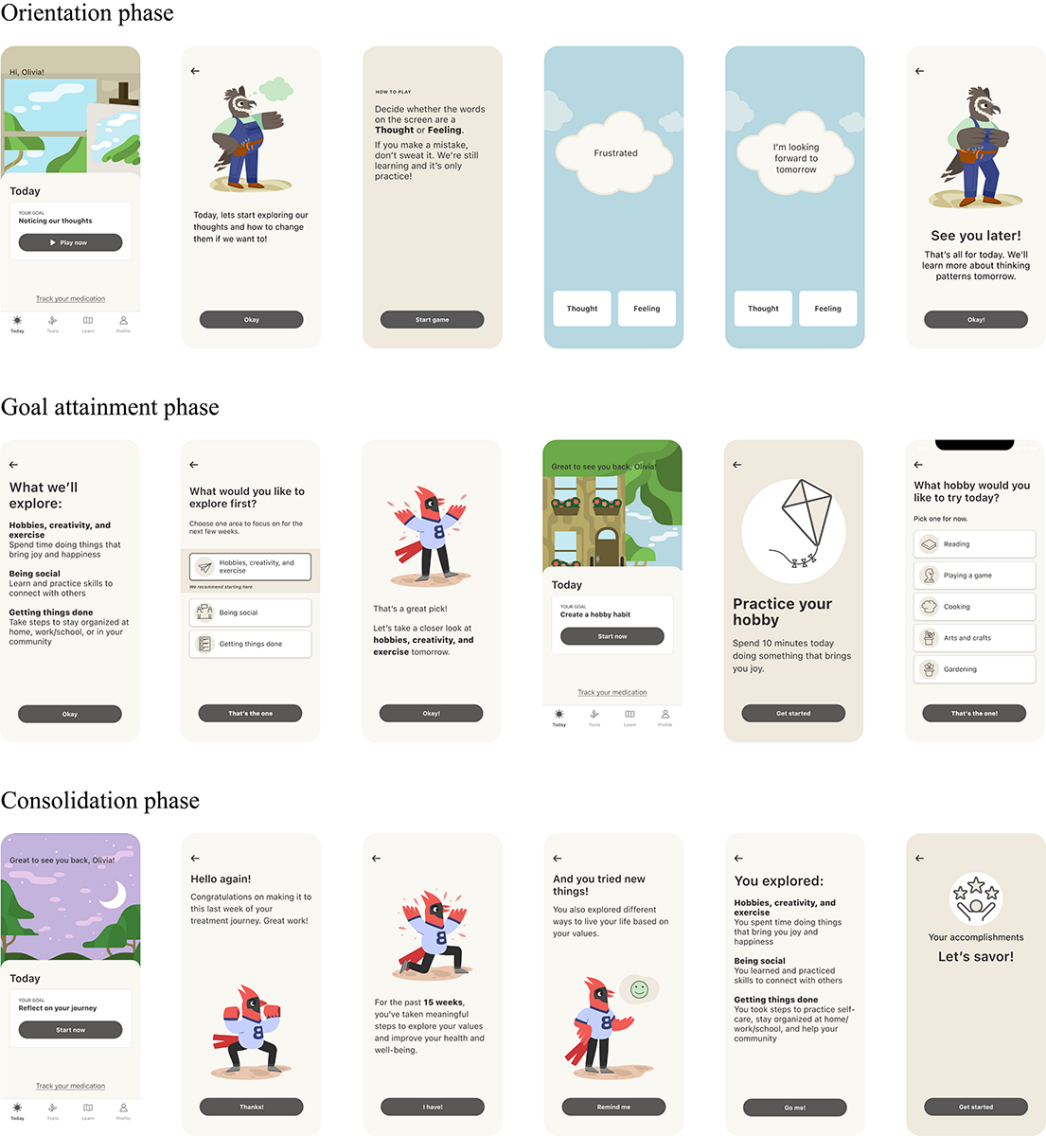
Example screenshots of the CT-155 study app representing foundational therapeutic skill as delivered during the orientation phase, adaptive goal setting during the goal attainment phase, and reflection and celebration of treatment progress during the consolidation phase. CT-155: CT-155/BI 3972080.

The components of CT-155 are designed to address defeatist beliefs and lack of motivation. Defeatist beliefs are a mediating variable between cognitive impairment, negative symptomatology, and poor functioning in schizophrenia [49], while a lack of motivation reduces an individual’s ability to successfully overcome the challenges of daily living with schizophrenia [87,88]. CT-155 components included the setting of goals appropriate to the patients’ current level of functioning (adaptive goal setting) [89,90] to promote real-world engagement (behavioral activation) [43,44], while targeting defeatist performance beliefs (cognitive restructuring) [47,49,72] and delivering therapeutic interventions to facilitate successful goal attainment (social skills training [33,41,48,50], positive affect training [45,46], distress tolerance skills [91]). These core components are intended to remediate dysfunctions in reward pathways, which contribute to reduced internal representations of behavioral goals and drive motivational impairments central to ENS [92-94]. In particular, targeted behavioral activation strategies followed by cognitive restructuring promote engagement in goal-directed behaviors, while also creating and maintaining associations between effort and positive affect in relation to those behaviors [42,95,96]. A selection of screenshots from the CT-155 study app are presented in Figures 3 and 4.

CT-155 design and development were informed by an iterative patient-centered approach from 156 people living with schizophrenia. For example, the mood check-in feature was refined based on feedback from individuals with lived experience. Initially designed as a simple, single-screen daily prompt, it evolved into a dynamic tool offering tailored feedback and coping strategies aligned with users’ reported mood. Feedback also informed how therapeutic content was structured, the tone and pacing of the app’s narrative, and improvements in usability and overall user flow. Further details are available in Figure S1 in Multimedia Appendix 1 and have been described by Snipes et al [81] and Goenjian et al [74].

The active period for CT-155 comprised 3 structured but personalized phases within the app: 3-week orientation phase, 12-week goal attainment phase, and 1-week consolidation phase. The orientation phase included lessons to set expectations and encourage daily adherence, provide schizophrenia-specific psychoeducation, and help build an effective therapeutic alliance (also referred to as a digital working alliance [81]), which is important for engagement with DTx. The goal attainment phase included adaptive goal setting, social skills training, and cognitive restructuring. The consolidation phase included guided reflection on, and celebration of progress, and establishment of a plan for continued application of skills learned over the course of the study in daily life. Lessons and activities were delivered through brief, daily interactive modules designed to be completed in under 10 minutes. These included a combination of text-based explanations, short exercises, and reflection prompts tailored to each phase. Participants also completed daily check-ins and were encouraged to access content regularly. The app included SMS and push notifications to prompt check-in completion and encourage daily lesson engagement. Two prior exploratory studies using earlier versions of CT-155 (CT-155 beta) demonstrated initial feasibility in engaging people living with schizophrenia and an associated reduction in ENS severity following 7 weeks of use [74,81].

### Digital Control

The digital control app served as a comparator that controlled for common elements of a digital app (eg, receipt of notifications, on-demand access), daily engagement, and face validity [78,97]. It was designed to be reasonably equivalent to the active treatment in terms of daily engagement and face validity. For example, the user interface was similar (Figure 3 depicts the identical log-in screens of the CT-155 app and the digital control app), and the experience included SMS text messages, push reminders, daily check-ins, and daily activities. This design aimed to promote frequent use of the app and ensure that any differences in outcomes could be attributed to the therapeutic content, rather than to nonspecific effects of digital engagement. The digital control app also included elements of the disease-specific educational components of CT-155. The educational components were designed to increase knowledge about schizophrenia without encouraging behavior change or providing other therapeutic benefits.

### Outcomes and Assessments

In line with FDA current best practices and terminology in medical device clinical research, improvement in clinical outcomes is herein referred to as effectiveness (rather than efficacy) [98].

End points included clinician-reported and patient-reported assessments. The primary effectiveness end point was change from baseline to Week 16 in ENS severity, as assessed by CAINS-MAP (Table 1). CAINS is a 13-item interview-based, clinician-administered assessment that consists of 2 subscales covering the 5 domains of negative symptoms: Motivation and Pleasure (referred to as “MAP”; ie, avolition, anhedonia, and asociality), and Expressivity (referred to as “EXP”; ie, blunted affect, alogia). CAINS items are scored on a 5-point scale, with lower scores indicating lower negative symptom severity [99]. CAINS is a second-generation negative symptoms scale that was developed to reflect updated conceptualizations of negative symptoms, as recommended by the National Institute of Mental Health and the Measurement and Treatment Research to Improve Cognition in Schizophrenia initiative [99,100]. CAINS offers a more detailed assessment of negative symptoms than older scales such as the Positive and Negative Syndrome Scale (PANSS) and assesses internal experiences (eg, joy, pleasure, interest, and intimacy) rather than just behavior to focus on experiential deficits important to the individual [101,102]. CAINS was administered by a centralized rater team. Raters were trained consistently with didactic training and posttests, and video scoring exercises; interrater reliability was measured every 6 months.

**Table 1:** Study end points.

End point and measure			Description
**Primary**			
	Change from baseline to Week 16 in ENS^a^, as assessed by CAINS-MAP^b^ [99]	CAINS is a 13-item interview-based assessment comprised of 2 subscales that measure the 2 factors of negative symptoms: Motivation and Pleasure (MAP) and Expressivity (EXP). CAINS items are scored on a 5-point scale, with lower scores indicating lower negative symptom severity. The MAP scale consists of 9 items that measure interest and engagement in motivated behavior as well as the experience of pleasure across social, vocational, and recreational domains. Each item is scored based on patient-reported behavior and experience.	
**Secondary**			
	Change from baseline in motivation and pleasure symptoms at Week 8, as assessed by CAINS-MAP [99]	See CAINS-MAP description above.	
	Change from baseline in expressive negative symptoms at Weeks 8 and 16, as assessed by CAINS-EXP^c^ [99]	See CAINS description above. The EXP scale consists of 4 items that measure verbal intonation (prosody), nonverbal expressivity (gestures, posture), facial expressivity, and speech output (alogia). Items are rated based on observation over the course of the interview.	
	Change from baseline in positive symptoms at Weeks 8 and 16, as assessed by PANSS^d^ [103]	Consists of 3 subscales containing a total of 30 symptom constructs. For each symptom construct, severity is rated on a 7-point scale, with a score of one indicating the absence of symptoms and a score of 7 indicating extremely severe symptoms.	
	Change from baseline in social functioning at Weeks 8 and 16, as assessed by PSP^e^ [104]	Validated clinician-rated scale that measures personal and social functioning in 4 domains: socially useful activities (eg, work and study), personal and social relationships, self-care, and disturbing and aggressive behaviors. Each area is scored on a 0-100 scale, with anchors for every 10-point interval.	
	Change from baseline in self-reported defeatist beliefs at Weeks 8 and 16, as assessed by DAS^f^ [105,106]	A 15-item subset of the DAS. Items are scored on a 1-7 scale, with higher scores indicating more severe defeatist thinking.	
	Change from baseline to Week 8 and to Week 16 in PGI-I^g^	A patient-reported outcome measuring subjective improvement in ENS severity on a 7-point scale. A higher PGI-I score indicates a subjective report of disease worsening over time.	
**Exploratory**			
	Engagement metrics	Measured passively as a result of participants’ day-to-day app use and completion of in-app activities. These were the number of days the app was used, the number of days with at least one app session ≥60 seconds, and the number of days with at least one daily activity completed.	
	Change from baseline in disease severity at Week 8 and 16, as assessed by CGI-S^h^ [107]	A standardized, clinician-rated global rating scale that measures ENS severity in the past 7 days using a 7-point Likert scale (1 being not ill at all; 7 being among the most extremely ill participants). A higher score on the CGI-S represents a higher severity of disease.	
	Change from baseline in disease severity at Week 8 and Week 16, as assessed by WHODAS 2.0^i^ [108]	A 36-item self-assessment scale to measure a participant’s function and disability across 6 domains of life: cognition (understanding and communicating), mobility (moving and getting around), self-care (hygiene, dressing, eating, staying alone), getting along (interacting with others), life activities (domestic responsibilities, leisure, work and school), and participation (community and society). Items are scored on a 5-point scale, with higher scores indicating greater disability.	
	Change from baseline in disease severity at Week 8 and Week 16, as assessed by SQLS-R4^j^ [109]	A 33-item self-assessment scale that assesses quality of life across domains of psychosocial feelings and vitality/cognition domains using a 5-point scale, with higher scores indicating comparatively lower quality of life.	
	Change from baseline to Week 8 and to Week 16 in EQ-5D-5L [110]	A standardized, brief self-report instrument for measuring health status. It consists of 2 components: the EQ-5D descriptive system and the EQ visual analogue scale. The descriptive system comprises 5 dimensions: mobility, self-care, usual activities, pain/discomfort, and anxiety/depression. The participant is asked to indicate his/her health status by selecting the box next to the most appropriate statement in each of the 5 dimensions. The EQ visual analog scale records the participant’s overall self-rated health on a visual analogue scale, from “the best health you can imagine” to “the worst health you can imagine.”	
	Change from baseline to Week 8 and to Week 16 in SDS^k^ [111]	A brief, 5-item self-report instrument that assesses functional impairment in 3 domains: work/school, social life, and family life. The first 3 items are rated from 0 (not at all) to 10 (extremely). There are 2 additional questions which measure the presenteeism and absenteeism over the preceding 7 days. The SDS total score is calculated as the sum of 3 items (range: 0-30), with higher scores indicating greater functional impairment.	
**Safety**			
	Frequency and severity of AEs^l^, serious AEs, and discontinuations from the study due to AEs Frequency and severity of AEs related to the worsening of positive or negative schizophrenia symptoms	Every event recorded in the form as “Adverse event” will be counted as an adverse event. Adverse events will be coded using the Medical Dictionary for Regulatory Activities (MedDRA).	
	Incidence of suicidal ideation and behavior, as identified via the C-SSRS^m^ [112]	A suicidal ideation and behavior rating scale that rates an individual’s degree of suicidal ideation on a scale, ranging from “wish to be dead” to “active suicidal ideation with specific plan and intent and behaviors.” Consists of a baseline evaluation that assesses the lifetime experience of the participant with suicide events and suicidal ideation, and a postbaseline evaluation that focuses on suicidality since the last study visit.	

^a^ENS: experiential negative symptoms.

^b^CAINS-MAP: Clinical Assessment Interview for Negative Symptoms Motivation and Pleasure subscale.

^c^CAINS-EXP: Clinical Assessment Interview for Negative Symptoms Expressivity subscale.

^d^PANSS: Positive and Negative Syndrome Scale.

^e^PSP: Personal and Social Performance Scale.

^f^DAS: Dysfunctional Attitudes Scale.

^g^PGI-I: Patient Global Impression of Improvement Scale.

^h^CGI-S: Clinical Global Impressions-Severity.

^i^WHODAS 2.0: World Health Organization Disability Assessment Schedule.

^j^SQLS-R4: Schizophrenia Quality of Life Scale – Revision 4.

^k^SDS: Sheehan Disability Scale.

^l^AE: adverse event.

^m^C-SSRS: Columbia-Suicide Severity Rating Scale.

Other study end points included change from baseline to Week 8 in ENS severity (assessed by CAINS-MAP), and change from baseline to Weeks 8 and 16 in expressive negative symptoms (assessed by CAINS Expressivity subscale [CAINS-EXP]), positive symptoms (PANSS), social functioning (Personal and Social Performance Scale), self-reported defeatist beliefs (Dysfunctional Attitudes Scale), Patient Global Impression of Improvement Scale, Clinical Global Impressions-Severity, and quality of life (Schizophrenia Quality of Life Scale – Revision 4). PANSS consists of 3 subscales (positive, negative, and general psychopathology) containing a total of 30 symptom constructs. For each symptom construct, severity is rated on a 7-point scale, with a score of 1 indicating the absence of symptoms and a score of 7 indicating extremely severe symptoms [99]. Further assessment details are described in Table 1.

Participant engagement with CT‑155 and digital control were measured passively by the smartphone as a result of participants’ day-to-day app use and completion of in-app activities. These included the number of days the app was used, the number of days with at least 1 app session ≥60 seconds, and the number of days with at least 1 daily activity completed. Daily activities in CT-155 included completing lessons, completing check-ins, practicing skills, or completing steps to attain goals. Daily activities in the digital control included completing lessons or completing check-ins. Participants were considered adherent during the study if they completed at least 1 daily activity for at least 67 of the 112 days of the active period (≈60%). The schedule of assessments is presented in Table 2.

**Table 2:** Schedule of activities and assessments.

Study day and visit window		Screening period	Active period						Follow-up period
		Screening^a^ (days −14 to −1 [minimum of 7 days between screening and baseline CAINS^b^])	Baseline (day 1)	Week 4 (day 28 ± 3 days)	Week 8 (day 56 ± 3 days)	Week 12 (day 84 ± 3 days)	Week 16/early termination (day 112 ± 3 days)	Week 20 (day 140 ± 3 days)	
Study visit number		1	2	3	4	5	6	7	
Clinic visit		✓^c^	✓		✓		✓		
Remote visit^d^				✓		✓		✓	
Informed consent		✓							
Demographics		✓							
Inclusion/exclusion criteria		✓	✓						
Medical history		✓							
Psychiatric history and *DSM-5*^e^ diagnosis of schizophrenia		✓							
Urine drug screen^f^		✓	✓		✓		✓		
Urine pregnancy test^g^		✓	✓		✓		✓		
Randomization			✓						
Download app and confirm understanding^h^		✓	✓						
Complete enrollment in CT-155 study app to activate the assigned intervention			✓						
Concomitant therapy		✓	✓	✓	✓	✓	✓	✓	
Adverse events		✓	✓	✓	✓	✓	✓	✓	
**Clinician-reported outcomes**									
	CAINS^i^	✓	✓		✓		✓		
	PANSS^j^	✓	✓		✓		✓		
	CGI-S^k^		✓		✓		✓		
	PSP^l^		✓		✓		✓		
	MINI^m^	✓							
	C-SSRS^n^	✓	✓	✓	✓	✓	✓		
**Patient-reported outcomes**									
	PGI-I^o^				✓		✓		
	PGI-S^p^		✓		✓		✓		
	DAS^q^		✓		✓		✓		
	EQ-5D-5L		✓		✓		✓		
	Sheehan Disability Scale (SDS)		✓		✓		✓		
	WHODAS 2.0^r^		✓		✓		✓		
	SQLS-R4^s^		✓		✓		✓		
Adherence check (use of study app)				✓	✓	✓			
Uninstall study app								✓	

^a^The screening period was a minimum of 7 days before the baseline visit to allow for proper CAINS assessments.

^b^CAINS: Clinical Assessment Interview for Negative Symptoms.

^c^Check marks show the activities and assessments that occurred at each study visit.

^d^All remote visits were conducted via telephone. The Week 20 visit could be in-person if enrolling in NCT06067984, an open label extension study, the purpose of which was to evaluate the maintenance of effect as well as the safety of a second consecutive course of CT-155 [113].

^e^DSM-5: Diagnostic and Statistical Manual of Mental Disorders (Fifth Edition).

^f^The urine drug screen was conducted on-site.

^g^The urine pregnancy test was conducted on-site using the dipstick method.

^h^The study app could be installed at screening visit or baseline visit.

^i^CAINS was administered by a centralized blinded rater team, not the site clinician.

^j^PANSS: Positive and Negative Syndrome Scale.

^k^CGI-S: Clinical Global Impressions-Severity.

^l^PSP: Personal and Social Performance Scale.

^m^MINI: Mini International Neuropsychiatric Interview.

^n^C-SSRS: Columbia-Suicide Severity Rating Scale.

^o^PGI-I: Patient Global Impression of Improvement.

^p^PGI-S: Patient Global Impression of Severity.

^q^DAS: Dysfunctional Attitudes Scale.

^r^WHODAS 2.0: World Health Organization Disability Assessment Schedule.

^s^SQLS-R4: Schizophrenia Quality of Life Scale – Revision 4.

Safety end points included the frequency and severity of adverse events (AEs) and serious AEs, including those related to the worsening of positive or negative schizophrenia symptoms. The incidence of suicidality, as identified via the Columbia-Suicide Severity Rating Scale was also assessed.

### Sample Size Determination

Using a 2-sample *t* test approach and assuming a type I error rate of 5% (2-sided), 173 participants per arm were required to achieve 90% power to detect an effect size of Cohen *d* of 0.35. Assuming 20% early termination, a total of 432 participants was calculated as being needed before being randomized to the study in a 1:1 ratio (approximately 216 in each arm). The assumed effect size (Cohen *d*=0.35) was informed by an earlier exploratory study of a related version of the digital therapeutic [74], early research studies with psychosocial interventions targeting negative symptoms [72,114,115], and moderate effect sizes seen in broader confirmatory trials in schizophrenia and major depressive disorder [116,117].

### Statistical Analyses

#### Overview

All effectiveness, demographics, and baseline characteristics analyses will be performed using the intent-to-treat (ITT) set, unless otherwise specified. Baseline and demographic characteristics, including age, sex, race, and ethnicity, will be summarized using descriptive statistics.

#### Primary End Point

The primary effectiveness end point of change from baseline to Week 16 in ENS will be analyzed using both the ITT and per-protocol populations, with the ITT population serving as the primary analysis and the per-protocol analysis as a supportive analysis. The primary analysis will be conducted using a mixed models repeated measures (MMRM) approach. Missing data will be handled by the MMRM model and will not be imputed separately [118]. Sensitivity analyses will also be performed for the primary end point in the ITT set by imputing missing data using a multiple imputation method based on missing-at-random assumption and by the tipping point method [119]. An alpha value of ≤.05 will denote a significant difference in the change from baseline at Week 16 between the 2 interventions and determine study success.

The primary end point will be repeated in the ITT set by the following prespecified subgroups: length of time since diagnosis of schizophrenia, baseline CAINS-MAP negative symptom severity, sex, age group, race, ethnicity, education level, income level, living status, employment status, and geographical region. These subgroups were selected based on their relevance to clinical variability and social determinants of health in schizophrenia, which may impact symptom presentation and response to intervention [120-124]. Subgroup analyses are exploratory and are intended to assess the consistency of treatment effects across these distinct populations. No multiplicity adjustment will be applied to subgroup analyses for the primary effectiveness end point; nominal *P* values will be reported and interpreted descriptively, in line with their supportive role in the analytic framework.

#### Secondary End Points

All secondary end points will be analyzed in a similar manner to the primary end point. The analysis is the difference between the interventions using the MMRM and conducted in the ITT set. These analyses are considered nonhierarchical and will be interpreted in a supportive, exploratory framework. This analytical approach reflects health authority guidance for clinical studies, which consist of 2 treatment groups, use a single primary variable, and have a confirmatory statistical strategy that prespecifies just 1 single null hypothesis relating to the primary variable [125-127]. There is no formal multiplicity control for the secondary end points. The results are intended to provide additional context to the primary end point and to inform future research directions.

#### Exploratory End Points

Key engagement metrics will be summarized descriptively by visit and by treatment group. All other exploratory end points will be analyzed using a similar MMRM method as for the primary end point by visit and treatment group.

#### Safety End Points

All safety end points will be analyzed in the safety analysis set, defined as all randomized participants who signed the informed consent form, activated the study app, and completed at least 1 available daily activity in the study app during the active period. Safety data will be summarized descriptively by treatment group. All AEs will be coded by system organ class and Medical Dictionary for Regulatory Activities preferred term. An independent Data Safety Monitoring Board reviewed safety events throughout the study period. Any technical issues reported by participants were assessed for potential AEs and forwarded to the Data Safety Monitoring Board for review.

## Results

The study started enrolling in March 2023. As of January 2025, study recruitment was complete, enrolling 457 participants across 66 clinical study sites.

## Discussion

### Principal Findings

The treatment of negative symptoms is a critical unmet need for people living with schizophrenia. Evidence-based DTx have demonstrated the potential to provide a scalable new patient-centered treatment option to enhance existing care for individuals with schizophrenia [63,74,81]. However, well-designed randomized clinical trials are essential to rigorously evaluate the effectiveness and safety of DTx, and to generate the evidence required for FDA regulatory approval. We summarized the clinical trial protocol for the CONVOKE study, the largest phase 3, randomized, controlled, double-blind trial conducted to date to assess the effectiveness and safety of a DTx in schizophrenia. CT-155 is an investigational adjunctive treatment for negative symptoms in people living with schizophrenia who are on standard-of-care antipsychotic medication.

The CONVOKE protocol included study design elements that strengthen study rigor. First, CONVOKE will evaluate the potential of CT-155 to reduce participants’ ENS of schizophrenia using the CAINS-MAP assessment. CAINS is a second-generation negative symptom scale recommended by experts as a more detailed measure of negative symptoms compared with the older PANSS assessment, capturing both experiential (CAINS-MAP) and expressive (CAINS-EXP) symptoms [99,100,128]. Compared with PANSS, CAINS demonstrates better construct validity and coverage of negative symptoms domains, better psychometric properties, and more sensitivity in detecting treatment-related changes [99,101,102,129]. Furthermore, CAINS-MAP was administered by central raters, an approach that has a significantly lower placebo affect than using site-based raters [130]. Second, the CONVOKE protocol was developed in the context of a conventional phase 3 registrational study, by including design aspects such as a blind-to-hypothesis and a digital control arm. The digital control app for CT-155 was designed to control for unintended therapeutic benefit that may arise from nonspecific factors (eg, self-monitoring, treatment expectations), referred to as a placebo response in pharmaceutical trials. To our knowledge, CONVOKE is the first DTx study in participants with negative symptoms of schizophrenia to include such a digital control. The blind-to-hypothesis approach requires the digital control app being masked as an active therapeutic by closely mirroring it in terms of design, delivery, participant engagement, and the duration of the intervention. Thus, the risk of unmasking and disengagement for those assigned to the digital control is expected to be reduced. However, it is important to examine whether the blind-to-hypothesis approach functioned as intended, and this could be investigated by comparing participant engagement between the 2 study arms.

Finally, in keeping with the FDA’s patient-focused drug development initiative, CT-155 development was informed by a patient-centered design approach that considered the needs of those living with schizophrenia [31]. Feedback was captured from peer support specialists with lived experience of schizophrenia and patient panel participants with a diagnosis of schizophrenia, to iteratively improve CT-155. This patient-centric approach also supports establishing the digital working alliance and optimizes participant experience by creating an interactive, empathic, knowledgeable, and personally meaningful content within the CT-155 app that is critical for treatment outcome. These robust study design elements lay the groundwork for strengthening study rigor and ultimately improving confidence in the effectiveness of CT-155.

### Safety

Safety analyses will determine whether CT-155 has a low safety risk and can be tolerated by participants, an important study design component for a prescription DTx to be made available under FDA regulation. CT-155 is expected to be well tolerated, as demonstrated in the 2 single-arm clinical learning studies [74,81]. In contrast to pharmacotherapy, DTx do not involve the administration of pharmaceutical compounds and are therefore not associated with side effects as frequently present for pharmaceuticals and may be considered minimal risk [131,132]. However, it is still important to assess the safety of a DTx throughout the study (eg, monitoring hospital admissions, referrals to crisis care, and suicidal ideation) [75,133-137]. The 4-week posttreatment follow-up was specifically designed to assess short-term safety and tolerability after treatment concluded, and was intended to monitor for adverse events, potential withdrawal effects, or other posttreatment concerns. To extend observation beyond the initial study and provide further insights into maintenance of effect and safety, there was an opportunity for some participants to enroll into an open-label extension study (NCT06067984) [113].

### Limitations

Although the protocol had strengths by utilizing a conventional phase 3 clinical study design, there were potential limitations that may have introduced selection bias that could limit the external validity and generalizability of findings. First, participants were required to be on a stable dose of antipsychotic medication (≤2 antipsychotics) for at least 12 weeks. This was intended to ensure stability and reduce the likelihood of symptom fluctuations which could impact engagement with digital intervention. However, this may have also excluded individuals with recent diagnoses or those who had started antipsychotics within the last 12 weeks. These groups may have different treatment needs, and future studies will be important to assess the potential benefits of this intervention in more acutely unwell or treatment-initiating populations.

To address broader generalizability beyond a confirmatory phase 3 trial population, there is an ongoing prospective cohort study (ENSPIRUS) that aims to assess the effectiveness and safety of CT-155 in a broader population (eg, no exclusion criteria for those who recently engaged in psychotherapy; NCT06791122) [138].

Second, participants were required to own a smartphone, with consistent internet access, and have stable housing. While necessary for evaluating a digital intervention, these criteria may introduce some selection bias towards participants with greater digital literacy, greater socioeconomic stability, or higher overall functioning which could limit the external validity and generalizability of findings to the broader population of people with schizophrenia. The requirement for smartphone ownership aimed to reduce nonspecific effects related to unfamiliarity with digital devices and to avoid variability in engagement that could arise if participants were using loaned devices rather than their own. Although smartphone ownership is increasingly common across all socioeconomic groups, including among individuals with serious mental illness, disparities in digital literacy, data affordability, and reliable internet access may still limit broad generalizability across the target population [64,65,139]. Future studies should consider hybrid models that include loaning smartphones to further access the impact of accessibility and inclusiveness.

Finally, as no correction for multiple comparisons was applied across secondary end points or subgroup analyses, there is an increased risk of type I error; thus, these findings should be interpreted with caution and considered supportive of the study’s primary objective.

### Conclusion

This manuscript describes the methodological design for CONVOKE, the largest robust pivotal clinical trial evaluating the effectiveness and safety of a DTx as an adjunct to standard-of-care antipsychotic medication for negative symptoms in people living with schizophrenia. CT-155 development was informed by a patient-centered design approach. The CONVOKE study design included the centrally rated CAINS with its CAINS-MAP subdomain as the primary end point, blind-to-hypothesis, and used digital control arm. The treatment of negative symptoms has been a critical unmet need for people living with schizophrenia and the results of this study may shift the treatment paradigm for them. If the pivotal trial data show a significant difference between CT-155 and the digital control, CT-155 would offer an accessible, evidence-based psychosocial treatment delivered through smartphones in a patient-centered manner to augment standard-of-care for people with negative symptoms of schizophrenia.

## Data Availability

To ensure independent interpretation of clinical study results and enable authors to fulfill their role and obligations under the ICMJE (International Committee of Medical Journal Editors) criteria, Boehringer Ingelheim grants all external authors access to relevant clinical study data. In adherence with the Boehringer Ingelheim Policy on Transparency and Publication of Clinical Study Data, scientific and medical researchers can request access to clinical study data, typically, 1 year after the approval has been granted by major Regulatory Authorities or after termination of the development program. Researchers should contact Vivli [140] to request access to study data and visit MyStudyWindow [141] for further information.

## Appendix

Multimedia Appendix 1 Eligibility criteria and an overview of the patient-centered design process used to create the CT-155 study app.

## Abbreviations

AE: adverse event
CAINS: Clinical Assessment Interview for Negative Symptoms
CAINS-MAP: Clinical Assessment Interview for Negative Symptoms Motivation and Pleasure subscale
CAINS-EXP: Clinical Assessment Interview for Negative Symptoms Expressivity subscale
CT-155: CT-155/BI 3972080
DSM-5: Diagnostic and Statistical Manual of Mental Disorders (Fifth Edition)
DTx: digital therapeutics
ENS: experiential negative symptoms
FDA: Food and Drug Administration
ITT: intent-to-treat
MMRM: mixed models repeated measures
PANSS: Positive and Negative Syndrome Scale
