# Supplementary material for: Effectiveness, Engagement, and Safety of a Digital Therapeutic (CT-155/BI 3972080) for Treating Negative Symptoms in People With Schizophrenia: Protocol for the Phase 3 CONVOKE Randomized Controlled Trial
Source: JMIR Res Protoc. 2025 Oct 7;14:e81293. doi: 10.2196/81293 (PMC12541272; doi:10.2196/81293)
Supplement: Multimedia Appendix 1 [file resprot_v14i1e81293_app1.docx]

**Supplementary material**

**Full eligibility criteria**

| **Inclusion**   1. Is willing and able to provide written informed consent to participate in the study, attend study visits, and comply with study-related requirements and assessments. 2. Adult or late adolescent, 18 years of age or older at the time of informed consent. 3. Fluent in written and spoken English, confirmed by ability to read and understand the informed consent form (ICF). 4. Has a primary diagnosis of schizophrenia using the diagnostic criteria for schizophrenia, as defined in the DSM-5, for at least 6 months prior to the screening visit. 5. Is in the stable phase of illness, as assessed by the investigator after review of medical records or documented discussion with the treating healthcare provider. Alternatively, the PI may make this determination, if necessary. The rationale for this determination must be documented in the patient source. 6. Has outpatient treatment status at the time of screening, with no inpatient treatment for schizophrenia within 12 weeks prior to screening. 7. Is on a stable dose of antipsychotic medication(s) for at least 12 weeks prior to randomization (Day 1), with dose adjustments permitted during the study as outlined within the respective package inserts of their current medication(s) as determined by the investigator. 8. Has obtained an average score of ≥2 (moderate to severe) in at least two of the three CAINS-MAP domains (Social, Work or Recreational) at the screening visit and at baseline (Day 1). 9. Is the sole user of an iPhone with an iPhone operating system (iOS) 14 or greater, or a smartphone with an Android operating system (OS) 10 or greater and is willing to download and use the specified study app required by the protocol. 10. Is willing and able to receive SMS text messages and push messages on their smartphone 11. Has access to an email address. 12. Has regular access to the Internet via cellular data plan and/or wi-fi. 13. Has stable housing and has remained at the same residence for at least 12 weeks prior to screening, with no anticipated housing changes during the duration of the study. 14. Understanding of and interest in the use of the study app during the screening period and the Baseline Visit (Day 1). |
| --- |
| **Exclusion**   1. Is currently treated with more than two (2) antipsychotic medications (including more than two dosage forms). 2. Has obtained a positive symptom item score of >4 (moderate) on P1-Delusions, P2-Disorganization, P3-Hallucinations, P6-Suspiciousness; or a positive symptom item score of >5 (moderate-severe) on items P4-Excitement, P5-Grandiosity; or a general psychopathology item score of >5 (moderate-severe) on items G1-Somatic concern, G3-Guilt feelings, G9-Unusual thought content, G10-Disorientation at the screening or baseline visit on the PANSS, thus indicating prominent positive symptoms. 3. Is currently receiving or has received psychotherapy, defined as individual or group-based structured treatment (e.g., cognitive behavioral therapy, social skills training, or vocational/occupational therapy) within 3 months (13 weeks) prior to screening per investigator assessment. 4. Meets DSM-5, for diagnoses not under investigation that will impact their compliance to the protocol, including schizophreniform, schizoaffective, or psychosis non-specific disorders (posttraumatic stress disorder [PTSD], bipolar disorder, major depressive disorder, or developmental disorders). 5. Meets criteria per DSM-5 for a current episode of depression, mania, or hypomania. 6. Has a DSM-5 diagnosis of moderate to severe substance abuse disorder (except tobacco use disorder) within the 12 months before screening (confirmed using the Mini International Neuropsychiatric Interview [MINI]), or current abuse as determined by urine toxicology screen. A screening subject with mild substance abuse disorder within the 12 months before screening must be discussed and agreed upon before he/she can be allowed into the study. 7. Has a positive UDS or participant self-reports use of synthetic cathinones (bath salts), synthetic cannabinoids (K2, Spice), inhalants, amphetamines (including MDMA/ecstasy), phencyclidine (PCP), cocaine, opiates, benzodiazepines, barbiturates, hallucinogens, or parenteral drugs. Participants with a positive urine drug test and/or recreational use of THC will not be excluded from the study at the discretion of the investigator. Participants with a positive UDS or self-report who have a valid prescription for barbiturates, benzodiazepines, or opiates will not be excluded from the study at the discretion of the investigator. 8. Participated in a CT-155 or CT-156 clinical study (including but not limited to CT-155-C-001, CT-155-C-002, CT-155-C-003, CT-155-D-001, CT-155-D-002, CT-155-D-003, CT-155-D-004 or CT-156-C-001, CT-156-C-002, CT-156-D-001); or has participated in a CT-155 user research study (CT-155-P-00x, CT-155-A-001). 9. Has participated in another interventional clinical study within the last 2 months or an observational study in the last 1 month. Potential participants whose data was used in a blinded retrospective chart review study will not be excluded from participation. 10. Currently needs or will likely require prohibited concomitant medications and/or therapy during the study, as determined by the investigator. 11. Has suicidal ideation or behavior, as assessed by the C-SSRS: 12. Participants with a “yes” response to either Items 4 or 5 on the C-SSRS Suicidal Ideation Item within the last 12 weeks prior to screening, or at the baseline visit 13. Participants with a “yes” response on the C-SSRS Suicidal Behavior Items within the last 6 months (26 weeks) prior to screening or at the baseline visit 14. Participants who, in the investigator’s opinion, present a serious suicide risk. |

**Figure S1.** Overview of patient-centered design process used to create the
CT-155 study app


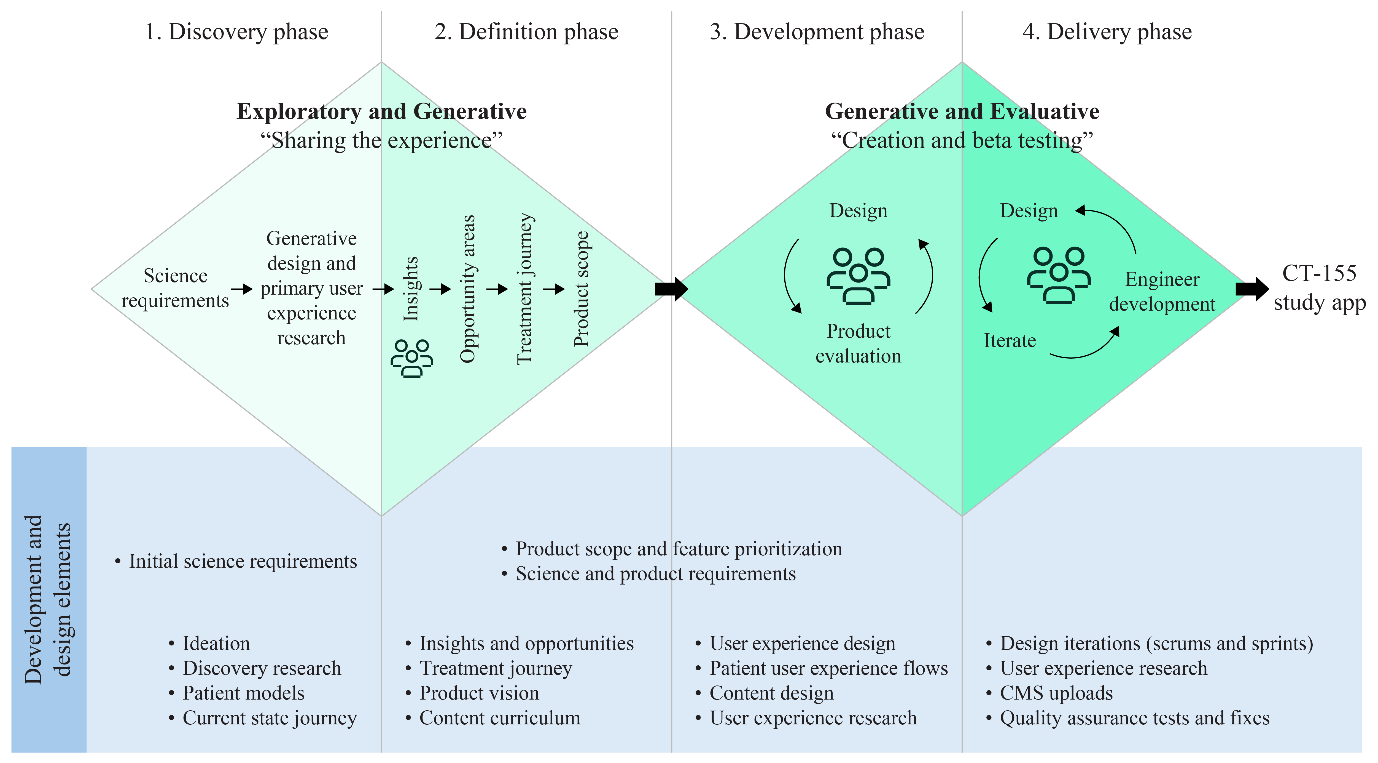


The exploratory and generative stage involved semi-structured interviews with 4 peer support specialists with lived experiences of schizophrenia, and 15 patient panel participants with a diagnosis of schizophrenia recruited through a patient-owned cooperative platform. The interviews were used to: 1) understand expectations of using a mobile app designed to treat symptoms of schizophrenia, 2) understand potential drivers and barriers to engagement with a digital therapeutic for someone diagnosed with schizophrenia, 3) collect feedback on concepts in development, including but not limited to content, narrative, information architecture, and usability. Concepts were presented using a combination of mock-ups and clickable prototypes, and interviews were conducted remotely using video-call platforms. Stage 2 was generative and evaluative and involved using the insights from Stage 1 to create beta versions of CT-155 for testing within 3 independent early-phase exploratory studies in people living with schizophrenia [1,2]. Participants from these early-phase studies completed a 1-hour exit interview after using the study app for 3 weeks (CT-155-C-001) and 7 weeks (CT-155-C-002; CT-155-C-003). The qualitative insights from these interviews helped understand: 1) experience using the study app, 2) drivers and barriers to engagement with the study app, 3) opportunities for usability and program journey improvements, 4) perceived value of specific therapeutic elements within CT-155 beta. In tandem with the study exit interviews, a combination of generative and evaluative semi-structured interviews continued to be conducted with the 4 peer support specialists and 15 patient panel participants, which were used to collect qualitative feedback on iterative concepts in development. Patients’ feedback from CT-155-C-001 helped informed the development/design of CT-155 beta for CT-155-C-002. All insights gathered helped further inform the development of the CT-155 study app.

CT-155 was developed in compliance with the relevant and applicable medical device standards and regulations. The product development processes utilized were in-line with current software development regulations, standards and guidance, and specifically are intended to manage risk and support the safety and effectiveness of the software app consistent with the principles described in International Electrotechnical Commission (IEC) 62304 consensus standard, “Medical device software - Software lifecycle processes”.

**References**

1. Snipes C, Dorner-Ciossek C, Hare BD, Besedina O, Campellone T, Petrova M, et al. Establishment and maintenance of a digital therapeutic alliance in people living with negative symptoms of schizophrenia: two exploratory single-arm studies. JMIR Ment Health. 2025 Jan 27;12:e64959. PMID: 39869902. doi: 10.2196/64959.

2. ClinicalTrials.gov. Exploratory study of a digital therapeutics in people with schizophrenia; Available from: <https://clinicaltrials.gov/study/NCT05438160>.
